# Comparative Evaluation of Convolutional Neural Network Object Detection Algorithms for Vehicle Detection

**DOI:** 10.3390/jimaging10070162

**Published:** 2024-07-05

**Authors:** Saieshan Reddy, Nelendran Pillay, Navin Singh

**Affiliations:** Department of Electronic and Computer Engineering, Durban University of Technology, Durban 4001, South Africa; trevorpi@dut.ac.za (N.P.); navins@dut.ac.za (N.S.)

**Keywords:** Convolutional Neural Networks, Object Detection, MATLAB, Faster R-CNN, YOLO, SSD

## Abstract

The domain of object detection was revolutionized with the introduction of Convolutional Neural Networks (CNNs) in the field of computer vision. This article aims to explore the architectural intricacies, methodological differences, and performance characteristics of three CNN-based object detection algorithms, namely Faster Region-Based Convolutional Network (R-CNN), You Only Look Once v3 (YOLO), and Single Shot MultiBox Detector (SSD) in the specific domain application of vehicle detection. The findings of this study indicate that the SSD object detection algorithm outperforms the other approaches in terms of both performance and processing speeds. The Faster R-CNN approach detected objects in images with an average speed of 5.1 s, achieving a mean average precision of 0.76 and an average loss of 0.467. YOLO v3 detected objects with an average speed of 1.16 s, achieving a mean average precision of 0.81 with an average loss of 1.183. In contrast, SSD detected objects with an average speed of 0.5 s, exhibiting the highest mean average precision of 0.92 despite having a higher average loss of 2.625. Notably, all three object detectors achieved an accuracy exceeding 99%.

## 1. Introduction

In the ever-expanding realm of computer vision, Convolutional Neural Networks (CNNs) have transformed the way machines comprehend and interpret visual data [1]. Central to this transformation is the task of object detection, a foundational concept with far-reaching implications across various fields including robotics, autonomous vehicles, surveillance, and healthcare [2]. CNN-driven object detection algorithms have emerged as the driving force behind significant progress in this domain, offering unmatched precision, efficiency, and scalability [3]. Object detection involves two primary goals: recognizing the presence of objects within an image and accurately pinpointing their spatial boundaries [4]. Traditional methods of object detection often relied on manually crafted feature extraction techniques followed by complex post-processing procedures. However, the rise of CNNs has brought about a paradigm shift, allowing for the direct learning of feature representations and spatial localization from data in an end-to-end manner [5]. CNN-based object detection algorithms typically employ a multi-stage approach, where initial candidate object regions, termed proposals, are generated and then refined through processes such as classification and bounding box regression. This pipeline is frequently enhanced with methods like region proposal networks (RPNs), anchor box mechanisms, and non-maximum suppression to boost accuracy and efficiency. Recent advancements in object detection have significantly benefited from the integration of various machine learning and deep learning techniques. The exploration of multi-view clustering with a novel manifold-based approach demonstrated enhanced performance in handling incomplete data [6], which is pertinent for robust object detection in complex environments. In the realm of domain adaptation, Wang et al. in 2013 [7] employed GAN-based scene synthesis to address domain shifts in video analysis, an approach that is particularly relevant for adapting object detection models across different video scenes. These methodologies align with the challenges faced in vehicle detection, where multi-view data integration and domain adaptation are crucial for accurate detection across varied scenarios. Additionally, recent studies in vehicle detection have utilized CNNs to achieve high precision and recall rates, underscoring the effectiveness of deep learning models in this domain [8,9,10]. This article provides a comparative analysis of three prominent CNN-driven object detection algorithms found in the literature, namely, Faster R-CNN, YOLO v3 (You Only Look Once), and SSD (Single Shot MultiBox Detector), focusing solely on their ability to detect vehicles.

**Figure 1:**
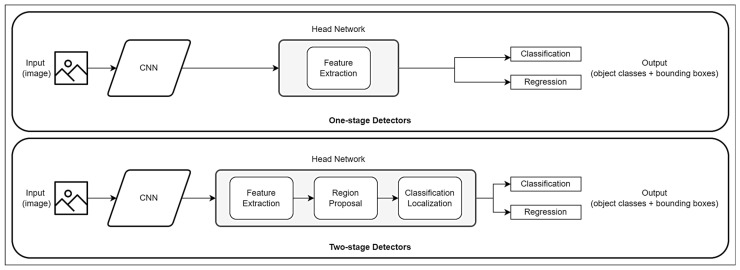
Architecture of one-stage detectors versus two-stage detectors.

Figure 1 illustrates the architecture of the two main detector categories, namely, one-stage detectors and two-stage detectors. Faster R-CNN belongs to the two-stage detector process whereas YOLO v3 and SSD form part of the one-stage detectors. The main difference between the two categories is that one-stage detectors can skip the step of region extractions and directly perform feature extraction, target classification and position regression within the network [11].

Proposed by Shaoqing Ren et al. in 2015 [12], Faster R-CNNs presents a two-step approach to object detection, which combines a Region Proposal Network (RPN) [13] with a Fast R-CNN [14] detector. The RPN functions as a fully convolutional network, operating on the feature map produced by a CNN backbone network such as VGG [15] or ResNet [16]. It generates proposals (bounding boxes) for potential objects by sliding a small window (referred to as an anchor) across the feature map and predicting objectness scores along with bounding box adjustments for each anchor. Once the RPN generates the region proposals, they are forwarded to the Fast R-CNN detector for object classification and refinement of bounding boxes. The Fast R-CNN detector utilizes RoI (Region of Interest) [17] pooling to extract features from each region proposal and then feeds them into fully connected layers for classification and bounding box regression. Since its inception, Faster R-CNN has catalyzed various advancements and extensions in object detection research [18,19]. Some notable developments include:

- Feature Pyramid Network (FPN): Integrating feature pyramid architectures with Faster R-CNN enhances the network’s ability to detect objects at multiple scales, improving performance on small and large objects alike.
- Attention Mechanisms: Incorporating attention mechanisms into Faster R-CNN allows the network to focus on relevant regions of the input image, leading to improved localization accuracy and robustness.
- Real-Time Implementation: Efforts have been made to optimize Faster R-CNN for real-time object detection applications, enabling efficient inference on resource constrained devices.

Introduced by Joseph Redmon and Ali Farhadi in 2018 [20], YOLO v3 expands upon earlier versions of YOLO by introducing several significant architectural enhancements. YOLO v3 employs a deeper convolutional neural network known as Darknet-53 [21] as its backbone. Darknet-53 is pretrained on the ImageNet dataset and acts as a feature extractor for identifying objects across multiple scales. YOLO v3 integrates a feature pyramid network to enhance object detection across various scales, improving its ability to detect smaller objects while maintaining high accuracy for larger ones. It predicts bounding boxes at three distinct scales, enabling the detection of objects of different sizes. Each scale is associated with a specific set of anchor boxes, which are optimized during training to enhance detection performance. Since its inception, YOLO v3 has sparked numerous advancements and extensions in the realm of object detection [22,23,24]. Some notable improvements include:

- YOLOv3-tiny: A lightweight version of YOLO v3 designed for real-time applications on resource-constrained devices.
- YOLOv3-spp: YOLOv3-spp (Spatial Pyramid Pooling) incorporates spatial pyramid pooling to improve feature representation and detection accuracy.
- YOLOv3-tiny-prn: An extension of YOLOv3-tiny that incorporates a partial residual network to enhance detection performance.

SSD, introduced by Wei Liu et al. in 2016 [25], is unique for its single-shot detection approach, which simultaneously forecasts object bounding boxes and class probabilities across multiple feature maps. SSD employs a foundational convolutional network (such as VGG or ResNet) to derive feature maps from input images [26]. These maps then undergo further processing by additional convolutional layers to anticipate bounding boxes and class scores at various spatial resolutions. SSD integrates feature maps at multiple scales to detect objects of diverse sizes. This is accomplished by employing convolutional layers with distinct kernel sizes and strides on the base feature maps, enabling SSD to capture objects at varying levels of detail. SSD forecasts bounding boxes using a set of default boxes, also referred to as anchor boxes, delineated at different aspect ratios and scales. These default boxes serve as standardized templates for predicting object positions and dimensions. Since its inception, SSD has spurred numerous advancements and extensions in the domain of object detection [27,28,29]. Some notable developments include:

- Improved Feature Extraction: Researchers have explored the use of advanced feature extraction techniques, such as FPN and residual connections, to enhance the representational power of SSD and improve detection performance.
- Efficient Backbones: Efforts have been made to develop lightweight backbone architectures tailored for SSD, enabling efficient inference on resource constrained devices without compromising detection accuracy.
- Domain Adaption: Techniques for domain adaptation have been proposed to improve the generalization capabilities of SSD across different environments and datasets, facilitating its deployment in real-world scenarios.

## 2. Methodology

The formulated dataset for this research was extracted from the following two datasets:

- Caltech Cars 1999: This dataset was created by Weber and Perona [30] which contained 126 images of cars from the rear with approximate scale normalization. The format of these images was in JPEG with a size of 896 × 592 pixels.
- Caltech Cars 2001: This dataset was created by Philip, Updike and Perona [31] which contained 526 images of cars from the rear with no scale normalization. The format of these images was in JPEG with a size of 360 × 240 pixels. This dataset included repeat images.

**Figure 2:**
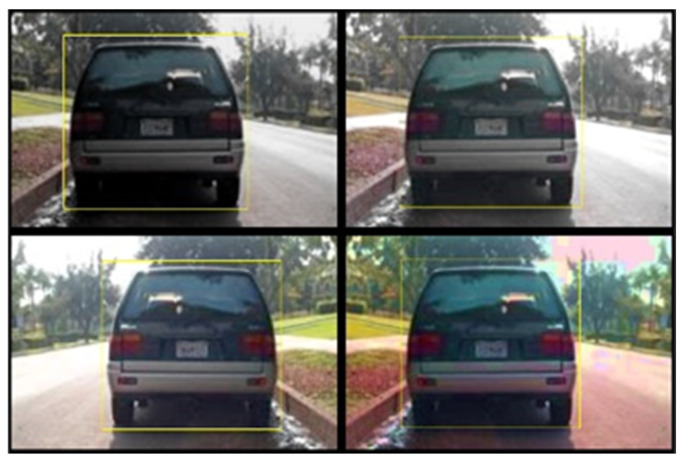
Result of data augmentation on a single image in MATLAB^®^.

The dataset sample used for this research contained a combined total of 652 images split into 391 images for the training dataset and 261 images for the test dataset. To decrease the computational cost of training object detection algorithms, the images underwent resizing before being processed. Additionally, to enhance the accuracy of the detector, random images underwent transformations via data augmentation. This technique enabled greater variety in the training data without requiring an increase in the number of labelled training samples. Using the *transform* function in MATLAB^®^, images were augmented by randomly flipping both the image and its corresponding box labels horizontally, adjusting the image’s scale, and introducing variations in color (brightness, contrast, and saturation). Figure 2 illustrates data augmentation in one of the images using the *transform* function. Algorithm 1 details the steps taken to augment and pre-process the images in MATLAB^®^. The *transform* function made use of the following functions:

- *randomAffine2d*: This created a randomized 2-D affine transformation and performed random horizontal reflection by parsing the *XReflection* argument.
- *imwarp*: This applied a geometric transformation to the image.
- *bboxwarp*: This applied a geometric transformation to the bounding boxes.

**Algorithm 1.** Data augmentation and pre-processing**Input:** Training image datastore 
**Output:** Transformed datastore 1.        Create an *ImageDatastore* for training images 2.        Read the images into the workspace to create image files 3.        **for each** image in the datastore **do** 4.                  Read each image 5.                  Randomly flip images and bounding boxes horizontally using *randomAffine2d*, *imwarp* and *bboxwarp* 6.                  Resize images and bounding boxes using *imresize* and *bboxresize* 7.        **end for**

**Figure 3:**
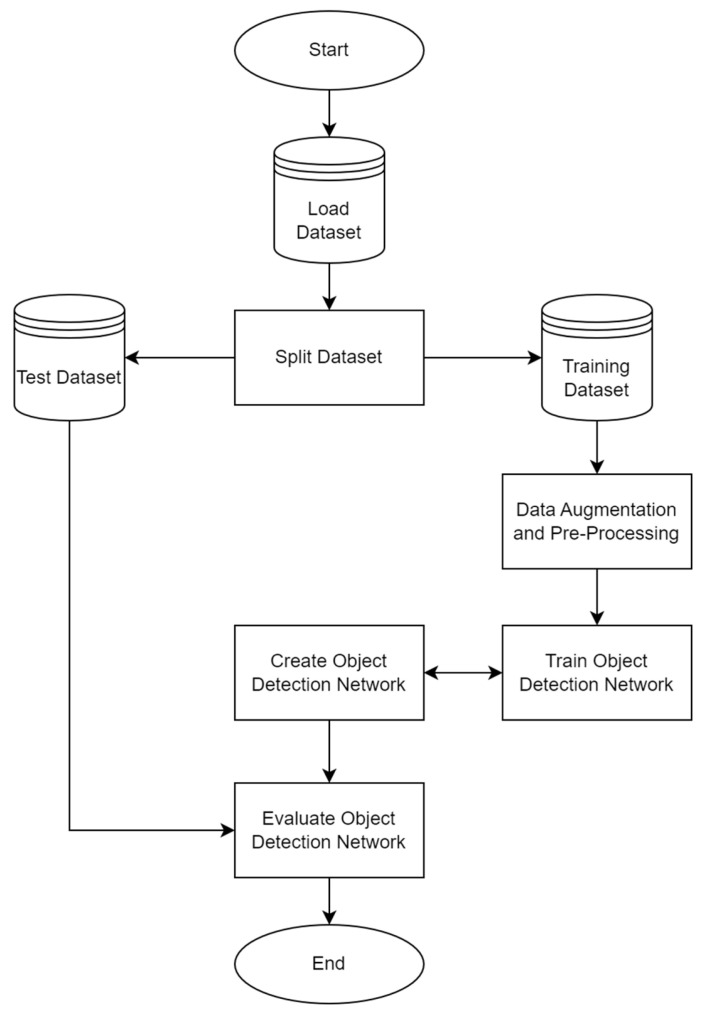
Steps in implementing object detection for each algorithm.

While the three object detection algorithms employ different techniques, the structure of the methods followed a similar approach. Figure 3 illustrates the main steps in implementing each of the object detection algorithms.

The Faster R-CNN model improves on the Fast R-CNN model, which in turn was expanded from the foundational R-CNN model. In the basic R-CNN model, a pretrained network serves as the preliminary point, while Fast R-CNN enhances object detection by incorporating a box regression layer, which learns a set of box offsets. Additionally, an ROI pooling layer is introduced to the network to pool CNN features for each region proposal. With Faster R-CNN, the Region Proposal Network (RPN) is integrated into the model to generate region proposals internally, eliminating the need for external algorithms to provide proposals. Equations (1) and (2) denotes the feature map subset for ROI pooling:

(1) $$F_{j}={RoI}_{pool}\left(F,\;R_{j}\right)$$

(2) $${RoI}_{pool}\left(i\right)=\sigma_{i}\;{RoI}_{pool}\left(i+1\right)+\left(\left(\frac{k_{i}-1}{2}\right)-p\right)$$

where *F* represents the feature map obtained from the backbone network, *R* represents the region proposals, and *σ* represents the Softmax function. The equation for the Softmax function is given as:

(3) $$\sigma {\left(z\right)}_{i}=\frac{e^{z_{i}}}{\sum_{j=1}^{K}e^{z_{j}}}$$

Typically, a pretrained CNN serves as the feature extraction network, with ResNet-50 being the chosen architecture for feature extraction in this study. MATLAB^®^ facilitates the automatic creation of a Faster R-CNN network through the *fasterRCNNLayers* function. Algorithm 2 provides details of the steps taken to manually develop the Faster R-CNN object detector in MATLAB^®^.
**Algorithm 2.** Faster R-CNNLoad a pretrained networkRemove the last 3 classification layersDefine and add new object classification layersDefine the number of outputs of the fully connected layerCreate and add the box regression layersConnect the regression layers to the *avg_pool* layerDisconnect the layers attached to the feature extraction layerAdd a ROI max pooling layerConnect feature extraction layer to ROI max pooling layerCreate the region proposal layerDefine the number of anchor boxes and feature mapsConnect to RPN to feature extraction layerAdd RPN classification and regression layersConnect the layers to the RPN network

The YOLO v3 model starts with a feature extraction network, serving as the foundational framework for the YOLO v3 deep learning network. While a pre-trained or untrained CNN can serve as the base network, transfer learning is only feasible with a pre-trained network. The creation of detection subnetworks involves the incorporation of convolution, batch normalization, and ReLU layers. The activation function, ReLU, is shown in Equation (4), where *x* is the input value:

(4) f(x)=max (0,x)

The detection network source comprises of the output layers that link to the detection subnetworks as inputs. In this study, the YOLO v3 object detector is established on SqueezeNet, utilizing the feature extraction network within SqueezeNet and integrating two detection heads at the end. MATLAB^®^ offers the capability to automatically generate a YOLO v3 network using the *yolov3ObjectDetector* function. Algorithm 3 details the steps taken to manually develop the YOLO v3 object detector in MATLAB^®^.
**Algorithm 3.** YOLO v3Load a pretrained networkInspect the base network architecture using *analyzeNetwork*Specify the anchor boxes and the classes to use to train the YOLO v3 networkSelect two feature extraction layers in the base network to serve as the source for detection subnetworkCreate the YOLO v3 detector by adding detection heads to the feature extraction layers of the base networkSpecify the model’s name, classes, and the anchor boxesInspect the YOLO v3 deep learning network using *analyzeNetwork*

The SSD model begins with a feature extraction network, which serves as the foundational framework for the SSD deep learning network. While a pre-trained or untrained CNN can be employed as the base network, transfer learning is only feasible with a pre-trained network. Prediction layers are chosen from the feature extraction network, in which any layer can be utilized. The outputs of these prediction layers are directed into classification and regression branches. The classification branches from all prediction layers are combined and linked to a Softmax layer, followed by a Binary Cross-Entropy (BCE) layer, which calculates the classification loss. The equation for BCE is given as:

(5) $$BCE\left(y,p\right)=-\sum_{i=1}^{n}{[y}_{i}\times \log\left(p_{i}\right)+(1-y_{i})\times \log\left(1-p_{i}\right)]$$

where *y* is the true label, and *p* is the predicted probability of the sample class.

Similarly, the regression branches from all prediction layers are consolidated and connected as a bounding box regression layer, which computes the bounding box loss. MATLAB^®^ provides the capability to automatically generate an SSD network using the *ssdObjectDetector* function. Algorithm 4 gives details of the steps taken to develop the SSD object detector in MATLAB^®^.
**Algorithm 4.** SSDLoad a pretrained networkDisplay the base network architecture using *analyzeNetwork*Specify the class names and anchor boxes to use for trainingSpecify the names of the feature extraction layers in the base network to use as the detection headsCreate the SSD object detector by using the specified base network and the detection heads

Mean Average Precision (mAP) stands as a cornerstone metric widely utilized for evaluating the performance of object detection algorithms. The computation of mAP entails several steps, including precision and recall calculations for each class, the construction of a precision-recall curve, and the averaging of precision across all classes. Precision is the capability of a model to only recognize significant objects, determined through True Positive (TP) and False Positive (FP) calculations. Recall, on the other hand, measures the proportion of relevant instances successfully retrieved, computed via TP and False Negative (FN) assessments. The Average Precision (AP) represents the performance metric derived from the area under the precision-recall curve. The respective performance measures are defined as:

(6) $$P=\frac{TP}{\left(TP+FP\right)}$$

(7) $$R=\frac{TP}{\left(TP+FN\right)}$$

(8) $$mAP=\frac{1}{N}\sum_{i=0}^{N}{AP}_{i}$$

## 3. Results

**Figure 4:**
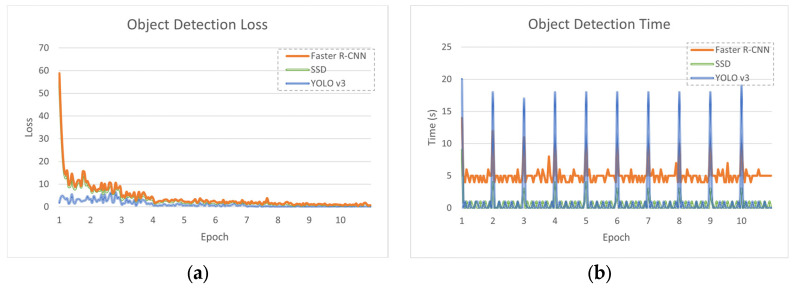
Training results observed for the three object detection algorithms at every epoch. (**a**) Object detection loss of Faster R-CNN, SSD and YOLO v3. (**b**) Object detection time of Faster R-CNN, SSD and YOLO v3.

Figure 4 illustrates the training results observed during this study. The Faster R-CNN model exhibited a decrease in average loss across epochs, starting at 1.453 in the initial epoch and reaching 0.105 in the final epoch. This decline signified the model’s effective learning from the training data, adjusting parameters to minimize loss and enhance performance. Notably, the most significant decrease occurred between the first and second epochs, suggesting rapid learning and substantial improvements. Despite fluctuations, the average detection time remained relatively stable, ranging from 4.9 to 5.3 s, indicating consistent computational efficiency throughout training.

In contrast, the SSD model began with a relatively high average loss of 11.992 in the first epoch, gradually decreasing to 0.807 by the ninth and tenth epochs. This trend indicated effective learning and improved predictive accuracy over time. Remarkably, the average detection time remained consistently low, ranging from 0.45 to 0.80 s, showcasing stable and efficient computational performance.

Similarly, YOLO v3 initially struggled with a high average loss of 3.202 in the first epoch, steadily decreasing to 0.080 by the tenth epoch. Notably, the most substantial reduction occurred between the first and fourth epochs, demonstrating rapid learning and significant improvements. The average detection time remained consistent throughout epochs, ranging from 1.10 to 1.30 s, highlighting stable computational efficiency.

**Figure 5:**
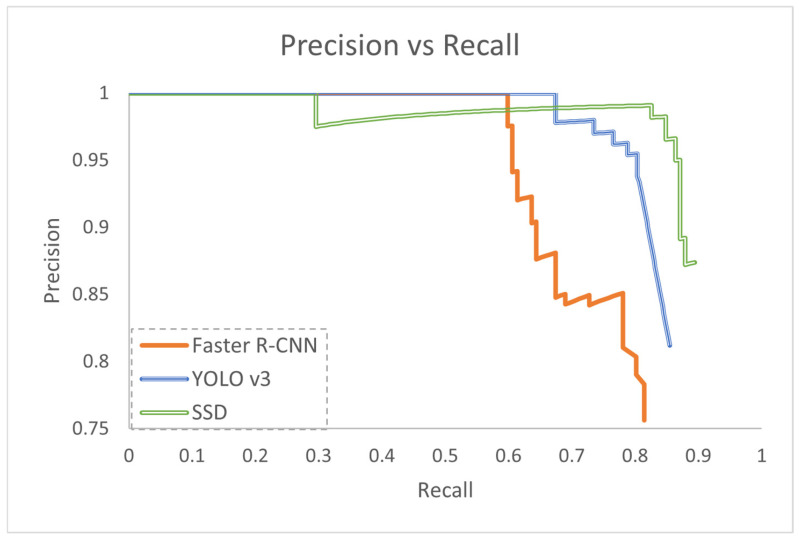
Precision versus Recall graph for the three object detection algorithms being evaluated.

Figure 5 illustrates the precision versus recall graph comparing the performance of Faster R-CNN, SSD and YOLO v3. For Faster R-CNN, precision remained consistently high at 1 for recall values ranging from 0 to 0.59, indicating accurate detections at low recall levels. However, as recall increased beyond 0.6, precision decreased marginally to ±0.9, suggesting the inclusion of false positives. Further increases in recall led to a noticeable decrease in precision, averaging around 0.8 between 0.7 to 0.8 recall. The precision-recall curve ended with a precision of 0.75 and a recall of 0.81, indicating a decrease in precision as the model aimed to capture more true positives. The model’s overall performance, evaluated using the mAP metric, yielded a value of 0.76, reflecting a balance between precision and recall across various object categories.

Similarly, SSD maintained ideal precision of 1 for recall values from 0 to 0.29, indicating accurate detections at low recall levels. Precision slightly decreased to 0.98 at a recall of 0.3, but gradually increased to 0.99 between 0.3 and 0.75 recall, showcasing the model’s ability to maintain high precision while detecting more objects. However, at higher recall values, precision dropped to 0.87, indicating a trade-off between precision and recall. The overall mAP value for SSD was 0.92, indicating a strong balance between precision and recall across different object categories.

In YOLO v3, precision remained at 1 for recall values from 0 to 0.67, suggesting accurate detections at low recall levels. However, as recall increased beyond 0.67, precision began to decrease, indicating the introduction of false positives. This decrease in precision continued as recall increased further, with a notable drop to 0.88 between 0.8 and 0.85 recall. The overall mAP value for YOLO v3 was 0.81, indicating a balance between precision and recall across various object categories.

**Figure 6:**
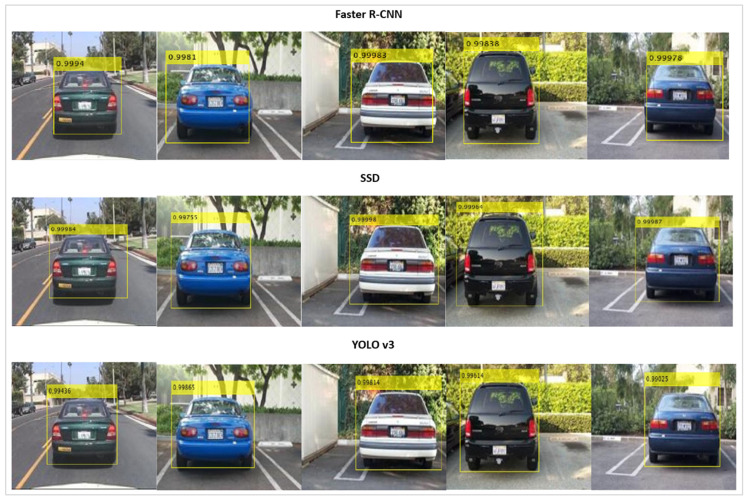
mAP results on a subset of images for Faster R-CNN, SSD and YOLO v3.

Figure 6 illustrates the mAP values for a subset of the dataset test images whilst using the Faster R-CNN, SSD and YOLO v3 object detector algorithms.

Table 1 summarizes the findings of this study. The SSD approach showcased the fastest average detection time among the three models, rendering it suitable for scenarios demanding real-time processing speed. YOLO v3 followed with moderate detection times, offering a balance between speed and precision. Conversely, Faster R-CNN presented higher detection times due to its two-stage methodology, where region proposals are generated before classification, making it less conducive to real-time applications but potentially enhancing accuracy. SSD displayed stabilized loss after an initial decline, while Faster R-CNN and YOLO v3 exhibited more varied trends. Despite fluctuations, all three models demonstrated notable average accuracy, with Faster R-CNN slightly edging out YOLO v3 and SSD. This reinforces their proficiency in accurately identifying objects in the dataset. SSD displayed the most consistent performance across epochs, maintaining stable detection time, loss, and accuracy. Conversely, while Faster R-CNN and YOLO v3 demonstrated stable accuracy, they exhibited minor fluctuations in detection time and loss.

## 4. Discussion

In the initial epochs of Faster R-CNN training, there were irregular peaks in loss, indicating high variability or instability in the training process. These spikes are typical in deep learning training and can result from factors such as random weight initialization or inadequate training data. However, as training progressed, the loss stabilized and consistently decreased, signaling the model’s convergence towards an optimal solution.

Similarly, in SSD, the first epoch exhibited a significant drop in loss, indicating rapid learning or adaptation to the training data. Subsequent reductions in loss suggested adjustments or optimizations during early training iterations, leading to improved performance.

In YOLO v3, erratic peaks in loss were observed in the first three epochs, indicating instability in the training process during initial iterations. Despite initial difficulties, the loss decreased significantly from the fourth epoch onwards, stabilizing around lower levels.

Additionally, notable peaks in detection time were observed for the first iteration in each epoch for all three object detection algorithms. This was primarily attributed to initialization processes such as loading data and initializing network weights, which demand additional computational resources and time compared to subsequent iterations.

Faster R-CNN, YOLO v3, and SSD all exhibited a common trend in their precision-recall curves. Faster R-CNN demonstrated high precision at lower recall values, attributed to its two-stage approach where proposals are generated before classification. However, precision decreased as recall increased, indicating challenges in maintaining accuracy with higher recall levels. Similarly, YOLO v3 showed high precision at lower recall values, followed by a gradual decline as recall increased. Despite its faster image processing capability owing to its single-stage architecture, YOLO v3 experienced a decrease in precision compared to two-stage methods such as Faster R-CNN. SSD also demonstrated high precision at lower recall values, followed by a gradual decline as recall increased. SSD’s design aimed to balance accuracy and speed by directly predicting object categories and bounding boxes from feature maps at various scales.

It is worth noting however that CNNs require large amounts of labelled data. In this research, a small dataset was useful and sufficient in exploring the different object detection training procedures, but in practice, more labelled images are needed to train a robust object detection network. Ground truth images also need to be manually segmented which can be time-consuming and is subjective. Future research directions may include establishing standardized benchmarks and evaluation protocols for comparing object detection models across different datasets. Further experimentation could also be carried out on datasets with multiple object classes and different programming implementations of the algorithms, such as Python, Java, and C++ to verify if the results presented in this study would be similar.

## 5. Conclusions

Under the conditions of the experiments carried out in this study, SSD emerged as the superior approach amongst the three algorithms, achieving the quickest average detection time of 0.5 s, the highest mAP of 0.92, and a commendable accuracy of 99.36%. Its streamlined design facilitates rapid and precise object detection, rendering it well-suited for real-time application in autonomous vehicles. YOLO v3 presents competitive performance, striking a balance between speed and accuracy, with a moderate detection time of 1.16 s and a relatively high mAP of 0.81. Its single-stage architecture ensures efficient image processing, lending itself to a variety of applications. Although Faster R-CNN achieves a competitive accuracy of 99.58% and a mAP of 0.76, its relatively longer average detection time of 5.1 s may restrict its suitability for being applied in autonomous vehicles. Nonetheless, its two-stage architecture enables precise object detection and classification, making it ideal for tasks prioritizing accuracy.

## Figures and Tables

**Table 1:** Image detection results for the three object detection algorithms.

Algorithm	Average Detection Time (seconds)	Average Loss	Accuracy (%)	mAP
Faster R-CNN	5.1	0.467 ^1^	99.58 ^1^	0.76
YOLO v3	1.16	1.183	99.47	0.81
SSD	0.5 ^1^	2.625	99.36	0.92 ^1^

^1^ Indicates best achieved result.

## Data Availability

The datasets used in this study are available in CaltechDATA at https://doi.org/10.22002/D1.20084 (accessed on 5 June 2024) and https://doi.org/10.22002/D1.20085 (accessed on 5 June 2024). The source code used in this study are openly available in GitHub at https://github.com/saieshan/object-detection-algorithms.git (accessed on 5 June 2024).
